# Repairing of N-mustard derivative BO-1055 induced DNA damage requires NER, HR, and MGMT-dependent DNA repair mechanisms

**DOI:** 10.18632/oncotarget.4514

**Published:** 2015-07-17

**Authors:** Ching-Ying Kuo, Wen-Cheng Chou, Chin-Chung Wu, Teng-Song Wong, Rajesh Kakadiya, Te-Chang Lee, Tsann-Long Su, Hui-Chun Wang

**Affiliations:** ^1^ Graduate Institute of Natural Products, College of Pharmacy, Kaohsiung Medical University, Kaohsiung 80708, Taiwan; ^2^ Institute of Biomedical Sciences, Academia Sinica, Taipei 11529, Taiwan; ^3^ PhD Program in Translational Medicine, College of Medicine/PhD Program in Toxicology, College of Pharmacy, Kaohsiung Medical University, Kaohsiung 80708, Taiwan; ^4^ Translational Research Center and Cancer Center, Kaohsiung Medical University Hospital, Kaohsiung 80756, Taiwan; ^5^ Department of Marine Biotechnology and Resources, National Sun Yat-Sen University, Kaohsiung 80424, Taiwan

**Keywords:** DNA repair, checkpoint, alkylation agents, DSBR, NER

## Abstract

Alkylating agents are frequently used as first-line chemotherapeutics for various newly diagnosed cancers. Disruption of genome integrity by such agents can lead to cell lethality if DNA lesions are not removed. Several DNA repair mechanisms participate in the recovery of mono- or bi-functional DNA alkylation. Thus, DNA repair capacity is correlated with the therapeutic response. Here, we assessed the function of novel water-soluble N-mustard BO-1055 (ureidomustin) in DNA damage response and repair mechanisms. As expected, BO-1055 induces ATM and ATR-mediated DNA damage response cascades, including downstream Chk1/Chk2 phosphorylation, S/G_2_ cell-cycle arrest, and cell death. Further investigation revealed that cell survival sensitivity to BO-1055 is comparable to that of mitomycin C. Both compounds require nucleotide excision repair and homologous recombination, but not non-homologous end-joining, to repair conventional cross-linking DNA damage. Interestingly and unlike mitomycin C and melphalan, MGMT activity was also observed in BO-1055 damage repair systems, which reflects the occurrence of O-alkyl DNA lesions. Combined treatment with ATM/ATR kinase inhibitors significantly increases BO-1055 sensitivity. Our study pinpoints that BO-1055 can be used for treating tumors that with deficient NER, HR, and MGMT DNA repair genes, or for synergistic therapy in tumors that DNA damage response have been suppressed.

## INTRODUCTION

DNA alkylating agents are frequently used in cancer chemotherapy. These agents are able to interact covalently (alkylation) with DNA, by forming mono- or bi-functional reactive intermediates. The mono-alkylating agents, such as temozolomide, are capable of transferring a single alkyl group to a DNA strand, resulting in DNA miscoding, strand breakage, cell death, mutagenesis, or carcinogenesis. The bi-functional alkylating agents, such as nitrogen mustard (N-mustard) derivatives (e.g., cyclophosphamide, chlorambucil, melphalan, carmustine, bis-chloroethyl), nitrosoureas (e.g., BCNU), platinum complexes (e.g., cisplatin), and mitomycin C (MMC) usually cause interstrand cross-linking (ICL). This kind of lesion blocks DNA replication and transcription. Thus, DNA alkylating agents are highly cytotoxic and are widely used as first-line adjuvant chemotherapeutics in treating various cancers. However, using DNA alkylating agents in chemotherapy has several drawbacks. This includes a drug's high chemical reactivity, resulting in the loss of therapeutic efficacy due to hydrolysis or in reactions with other cellular components [1, 2], lack of intrinsic DNA binding affinity that leads to carcinogenicity or bone marrow toxicity [3], and reduction of cytotoxicity via DNA repair mechanism [4] and acquired drug resistance (multi-drug resistance, MDR). Developing new DNA alkylating agents to improve existing problems is necessary to meet the clinical needs.

In vertebrates, different DNA repair machineries are developed in cells that are activated by various DNA-damage agents, and in turns protect against from these agents induced DNA lesions. Tumors with certain DNA repair gene deficiencies initially respond well to an appropriate DNA-damaging agent, but eventually develop resistance due to compensation by another DNA repair pathway [5]. Despite many resistance mechanisms having been discussed [6], the traditional chemotherapy regimens based on combinations of multiple DNA targeting agents are still currently most effective [7, 8]. One main reason is that multi-drug combinations produce diverse types of DNA lesions, overcoming rapid resistance to a single DNA-damaging agent. Typically, cells respond to DNA-damaging agents by activating the DNA damage response (DDR) pathway, which is a complex signal transduction cascade initiated by damage sensors, involving both transducers and effectors to maintain genome integrity. Following DNA damage, ataxia telangiectasia mutated (ATM) and the ATM- and Rad3-related (ATR) kinases act as two major DNA lesion sensors. They phosphorylate and activate the downstream effector checkpoint kinase 2 (Chk2) and 1 (Chk1), respectively, as a consequence of cell-cycle arrest, resulting either in the restoration of lesions or the elimination of unrecoverable cells through programmed cell death [9].

Several DNA repair mechanisms have been highly conserved throughout evolution. These mechanisms include 1) direct enzymatic repair by alkB homologs (ALKBHs) or by O^6^-methylguanine-DNA methyltransferase (MGMT), both of which can repair alkylated single-stranded DNA and RNA [10], and 2) the repair pathways, such as base excision repair (BER), nucleotide excision repair (NER), homologous recombination (HR), and non-homologous end-joining (NHEJ) [11]. For example, NER and HR are the most recognized repair mechanisms in response to melphalan-induced (an N-mustard) DNA damage. It is known that NER genes are required to remove N-alkylpurine adducts [12–14], and an extension of ICL repair capacity through an increase in HR and Fanconi's anemia (FA) protein expression was found to be involved in melphalan resistance [15–17]. These data not only provide information about the types of DNA lesions induced by melphalan, but also suggest the therapeutic implications of using the drug. Tumors with deficiencies in repair proteins should be hypersensitive to the corresponding chemotherapy. For example, tumors with low or no MGMT expression are highly sensitive to BCNU, which predominantly produces O^6^-alkylation adducts and can be removed by MGMT [10]. Therefore, understanding the response of the DNA repair system to lesions induced by DNA-damaging agent is critically important to the proper use of this agent in treating appropriate tumors with specific DNA repair gene defects.

Synthetic N-mustard BO-1055 has been previously found to produce plasmid DNA cross-linking damage and to exhibit anticancer activity *in vitro* and *in vivo* [18, 19]. In this study, we confirm that BO-1055 induces G_2_/M and S checkpoint arrest and apoptosis in cancer cells, and that both HR and NER are required for the removal of the DNA damage it induces, further supporting that BO-1055 causes DNA-ICL damage just like most of N-mustards do. For a comprehensive understanding of the effectiveness of BO-1055, we also examined the other DNA repair machineries, besides NER and HR, which are required for BO-1055 damage. Intriguingly, cells lacking MGMT activity, but not N-methylpurine-DNA glycosylase (MPG) or alkylated DNA repair protein AlkB homolog 2 (ABH2), were sensitive to BO-1055 treatment, revealing an as yet uncharacterized activity. These results suggest that the DNA repair process following BO-1055-induced lesions requires the involvement of NER, HR, and MGMT repair. These findings provide new insight into the clinical implications of BO-1055 treatment.

## RESULTS

### Repair of BO-1055-induced DNA damage requires HR and NER

**Figure 1:**
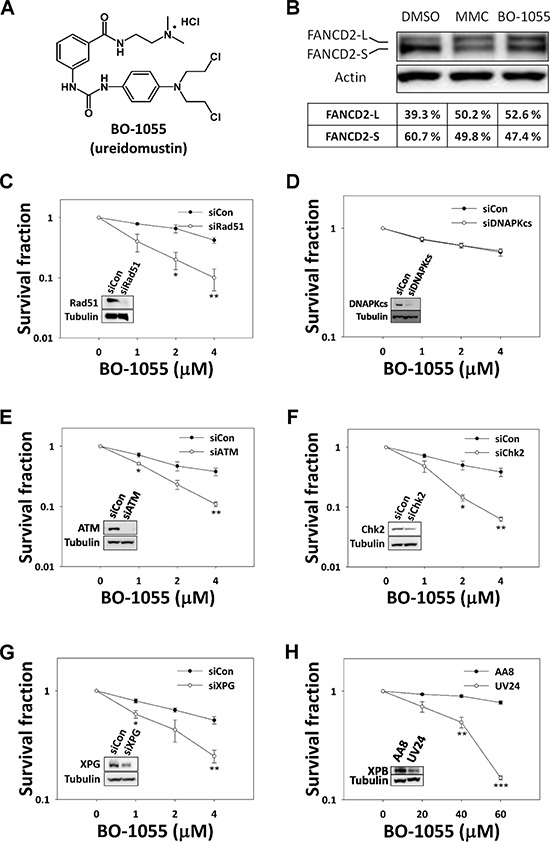
HR and NER genes are required to repair BO-1055 ICL lesions **A.** Chemical structure of BO-1055. **B.** Immunoblot analysis showing FANCD2 mono-ubiquitination following the exposure of MCF-7 cells to 5 μM of MMC or of BO-1055 for 6-h. FANCD2 (S-form) and mono-ubiquitinated FANCD2 (L-form) were detected using an antibody against FANCD2, and quantified using the Multi-Gauge software, V3.0 (Fujifilm). *In vitro* clonogenic survival of MCF-7 cells with knockdown of Rad51 **C.** DNA-PKcs **D.** ATM **E.** Chk2 **F.** or XPG **G.** by siRNAs, or of *XPB*-defective UV24 CHO cells **H.** exposed to the indicated doses of BO-1055 for 6-h. The immunoblots embedded in the clonogenic survival plots show the efficiency of gene knockdown for each individual experiment.

As BO-1055 (Figure 1A) has been recognized as a DNA-ICL inducer *in vitro* [19], we assessed whether DNA repair pathways corresponding to the removal of DNA-ICL are a required response to BO-1055 treatment. It was reported that, when DNA polymerases were stalled at the site of ICL during DNA replication, FANCD2 would be mono-ubiquitinated by FANCL, a FA-associated E3 ubiquitin ligase that is required for the efficient removal of ICL by homologous recombination repair. An evaluation of the fold change of non-ubiquitinated and mono-ubiquitinated FANCD2 at the molecular level is frequently adapted to monitor DNA-ICL damage [20]. As expected, the amount of mono-ubiquitinated FANCD2 (FANCD2-L) increased on treatment with BO-1055or MMC (Figure 1B), suggesting that either BO-1055 or MMC can induce chromosomal DNA-ICL that requires the FANCD2-mediated DNA repair pathway. In addition, as it has been reported that DNA-ICL can be repaired by double-strand break repair (DSBR) and NER proteins [21, 22], we examined whether cells were sensitive to BO-1055 when DNA repair gene expression was knocked down, or when carrying a DNA repair gene defect. To test the involvement of DSBR, we compared the BO-1055 sensitivity in MCF-7 with the knockdown of key players in HR and NHEJ, the repair protein Rad51 recombinase (Figure 1C) and the DNA protein kinase catalytic subunit (DNA-PKcs) (Figure 1D), respectively. We also knocked down the key DSB-corresponding checkpoint proteins, ATM (Figure 1E) and Chk2 (Figure 1F). The results show that the silencing of the expression of Rad51, ATM, or Chk2, but not DNA-PKcs, increases BO-1055 sensitivity, suggesting that BO-1055 DNA-ICL processing might produce DSB intermediates that require repair by HR, rather than by NHEJ. The involvement of NHEJ was also confirmed by pharmacological inhibition of DNP-PKcs by selective inhibitor NU7441 that cells incubating with NU7441 were more sensitive to doxorubicin but not BO-1055 treatment (Supplementary Figure S1A). A similar requirement of HR was also observed in Rad51 knockdown MCF-7 cells treated with MMC, which produce DNA-ICL that are well known to be repaired by the HR pathway (Supplementary Figure S1B). The structure-specific endonuclease xeroderma pigmentosum complementation group G (XPG) is an indispensable core protein in the NER pathway, and it has been linked to MMC lesion repair [23]. We knocked down XPG expression using small interfering RNA (siRNA), to test the involvement of NER, and the results showed that the silencing of XPG expression increases cell sensitivity to BO-1055 (Figure 1G), suggesting that NER is involved in repairing damage caused by BO-1055. Moreover, the UV24 cells, which are deficient in the xeroderma pigmentosum complementation group B (XPB), another protein involved in NER [24], were also sensitive to BO-1055 when compared to parental AA8 cells (Figure 1H). The requirement of NER was also observed in XPG knockdown MCF-7 and UV24 CHO cells treated with MMC (Supplementary Figure S1C and S1D).

### BO-1055 induces the DNA-damage response and perturbs cell cycle progression

**Figure 2:**
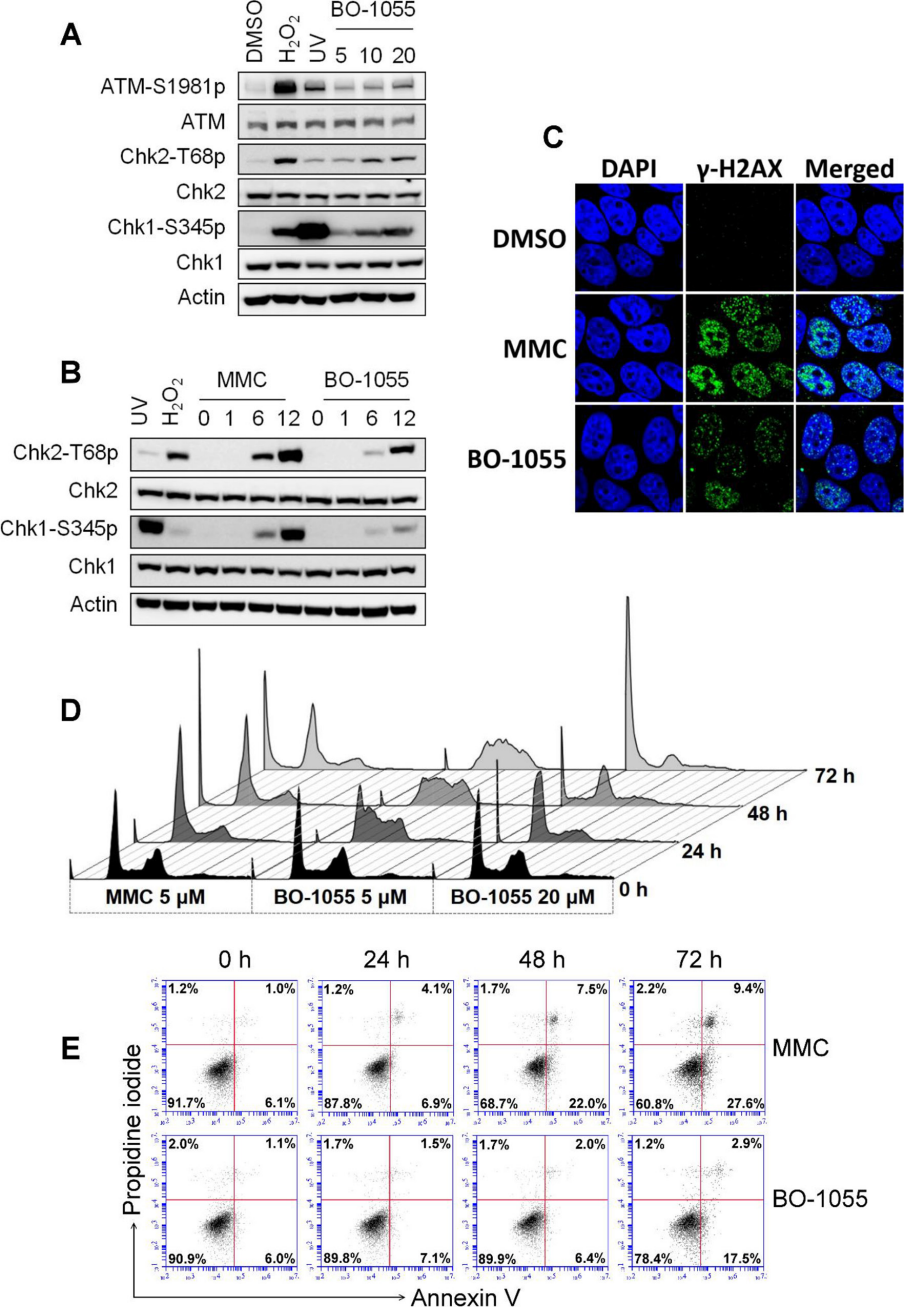
BO-1055 induces DDR and cell death **A.** Immunoblots showing DDR through the detection of the phosphorylation of ATM Ser1981(ATM-S1981p), Chk1 Ser^345^ (Chk1-S345p), or Chk2 Thr^68^ (Chk2-T68p), following the exposure of MCF-7 cells to 5, 10, or 20 μM of BO-1055 for 6-h. Cells treated with 0.1 mM of H_2_O_2_ and 10 J/m^2^ of UV for 30 min served as positive controls. **B.** The same experiment described in (A), cells were exposed to 5 μM of MMC or of BO-1055 for 0, 1, 6, or 12 hours. **C.** Immunohistochemical staining for the DNA damage marker γ-H2AX (green) and nucleus DAPI (blue) of cultured MCF-7 cells was conducted following incubation with 5 μM of MMC or BO-1055 for 24-h. **D.** FACS histogram analysis of DNA content. PI staining in fixed cells was performed following the exposure of cultured MCF-7 cells to the indicated doses of MMC or BO-1055 for the indicated times. **E.** FACS dot-blot analysis for cell death. AnnexinV/PI double staining in living cells was conducted following the exposure of cultured MCF-7 cells to 5 μM of MMC or of BO-1055 for the indicated times. The experiment of (D) and (E) were performed three times, and the quantitative results expressed as the mean ± SEM are respectively presented in Supplementary Figure S2A and S2B. The cell death, assessed in cells treated with 20 μM of MMC or of BO-1055, is presented in Supplementary Figure S2C.

Alterations in the chromatin architecture lead to the activation of ATM/ATR-mediated DDR pathways. As BO-1055 has been reported to crosslink to plasmid DNA *in vitro* [19] and requires NER and HR for repair, treating cells with it should elicit the DNA damage response. To confirm that, MCF-7 cells were subjected to BO-1055 treatment for 6-h. We found that the DNA damage-induced phosphorylation of Chk1-S345, Chk2-T68, and as well as that of ATM-S1981, increased in a dose-dependent manner (Figure 2A). It was also confirmed that BO-1055 induced DDR in a time-dependent manner starting 6-h following treatment early at 6-h (Figure 2B). However, the dose response to BO-1055 at 20 μM seemed to less effective than that to MMC at 5 uM, suggesting that BO-1055 is not as efficient as MMC in inducing DNA damage in MCF7 cells. We further analyzed the formation of γ-H2AX nuclear foci, a DNA-damage marker, to confirm this phenomenon, and found that BO-1055 induced fewer γ-H2AX nuclear foci than MMC (Figure 2C). As BO-1055 was found to induce ATM/ATR-dependent DNA damage checkpoint activation, we were interested in understanding the consequence of BO-1055 exposure at the cellular level. Flow cytometric analyses of the DNA content showed that MMC was able to induce S-phase cell-cycle arrest at dose of 5 μM for 24-hours of exposure, and the S-phase population continually accumulated while both the sub-G_1_ and polyploidy populations increased at 72-h. Even the same low dose of BO-1055 was found to accumulate in cells at the S and G_2_/M phase following a 48-h exposure, and to persist for up to 72-h, suggesting that BO-1055 and MMC have different ways to interact with DNA. However, a high dose of 20 μM of BO-1055 led to a rapid accumulation of both the S-phase following a 24-h exposure, while increasing the sub-G_1_ fraction 72-h later (Figure 2D, and Supplementary Figure S2A). The annexin V/PI-double staining assay was performed to characterize cell death. We found that 5 μM of MMC increased average of the annexin V-single-positive early apoptotic population from 11% to 26% following a 48-h exposure, and to 28% at 72-h. This exposure period also led to an increase average of a small portion of double-positive late apoptotic cells from 3% to 12%; 5 μM of BO-1055 caused a lesser effect, but significantly increased the early apoptotic cell population from 9% to 24% at 72-h (Figure 2E and Supplementary Figure S2B). This result suggests that treatment of MCF-7 cells with BO-1055 or MMC at a dosage of 5 μM, induces major apoptotic death. However, a high dosage of MMC and BO-1055 at 20 μM rapidly increased the PI-positive MCF-7 cells, suggesting that high doses of drugs causes the same level of cell toxicity and induces major necrotic-like death (Supplementary Figure S2C). The intensity of cell death induced by BO-1055, and that induced by MMC, as evaluated by the annexin V/PI-double staining and the MTT cytotoxicity assays (Supplementary Table S3), are mutually inclusive.

### MGMT is required for BO-1055-induced DNA lesions

**Figure 3:**
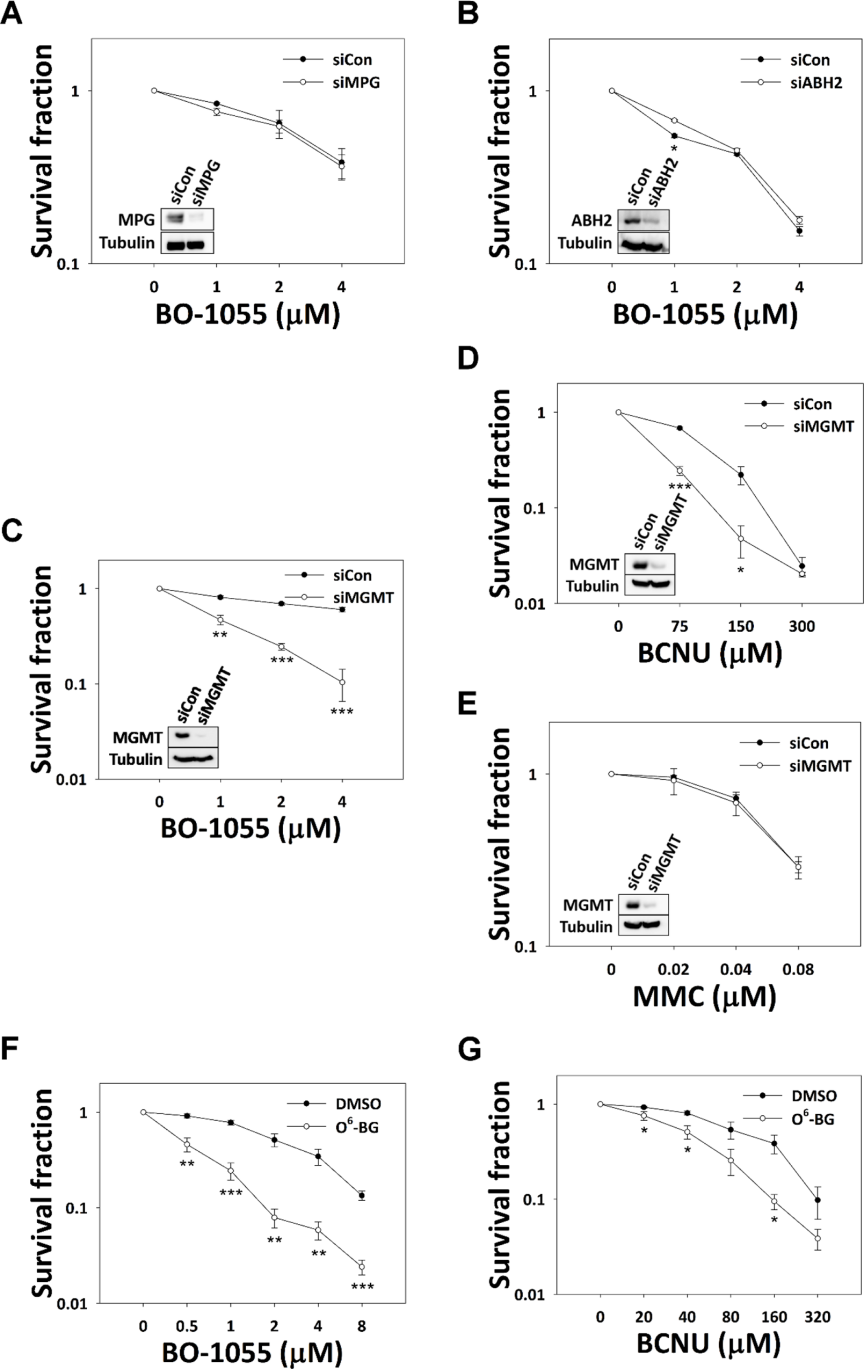
The involvement of base modification repair genes in BO-1055 lesions *In vitro* clonogenic survival of MCF-7 cells with knockdown of MPG **A.** ABH2 **B.** or MGMT **C.** by siRNAs, exposed to the indicated doses of BO-1055 for 6-h; knockdown of MGMT in MCF-7 cells exposed to the indicated doses of BCNU **D.** or MMC **E.** for 6-h was also performed. The immunoblots embedded in the clonogenic survival plots show the efficiency of gene knockdown for each individual experiment. The correlation of XRCC1 with BO-1055 sensitivity and the positive control for MMS damage in each set of conditional cells is listed in Supplementary Figure S3. *In vitro* clonogenic survival of MCF-7 cells, following inhibition of MGMT activity by 20 μM of O^6^-BG, in MCF-7 cells exposed to the indicated doses of BO-1055 **F.** or BCNU **G.** for 6-h.

As BO-1055 treatment led to DNA-ICL and DNA double strand breaks, the bi-functional alkylation of BO-1055 was explored; however, the ability of BO-1055 to generate the other forms of DNA damage, i.e. mono-adducts, has not yet been explored. BO-1055 treatment has a different impact on cell-cycle distribution and cell death, implying that some BO-1055 lesions are different compared to those induced by MMC. To test this presumption, we examined whether the cell sensitivity to BO-1055 depends on MPG, a protein of the BER pathway that repairs N^7^-guanine adducts induced by N-mustards [25]. Knockdown of MPG expression in MCF-7 cells by siRNAs was not sensitive to BO-1055 (Figure 3A). This result was also confirmed by the knockdown of a scaffold protein in the BER pathway, X-ray repair cross-complementing protein 1 (XRCC1) [26], which was not found to be sensitive to BO-1055 (Supplementary Figure S3A). Comparing BO-1055 sensitivity between XRCC1-proficient AA8 and XRCC1-deficient EM9 CHO cells led to similar results (Supplementary Figure S3B). Our results suggest that the BER pathway is not involved in BO-1055 DNA damage repair. ABH2 is a demethylase, primarily responsible to repair N^1^-adenine and N^3^-cytosine DNA methylation [27]. Knockdown of ABH2 expression by siRNAs did not alter BO-1055 sensitivity in MCF-7 cells (Figure 3B), suggesting that ABH2 is dispensable in BO-1055 DNA damage repair. However, the sensitivity to the mono-functional alkylating agent methyl methanesulfonate (MMS) was significantly increased in EM9 CHO cells (Supplementary Figure S3C) and in cells in which MPG, ABH2, and XRCC1 expression was knocked-down, respectively, by siRNAs (Supplementary Figure S3D). These data suggest that lesions produced by MMS, but not by BO-1055, require the ABH2 and BER repair pathways; these two agents indeed cause differential effects on genomic DNA. BO-1055 does not produce significant N-alkyl modifications on DNA bases; it only accounts for a small proportion of modifications, if any, that are insufficient to cause cell death.

Given that MGMT is an O-alkyl-related DNA methyltransferase that differs in its function from ABH2 and MPG in N-alkyl base lesions, the involvement of MGMT in BO-1055 damage repair was examined. We found that knockdown of the expression of MGMT by siRNAs increased the sensitivity of MCF-7 cells to BO-1055 (Figure 3C), as well as that to BCNU (Figure 3D), which has been recognized as a one of the DNA O-alkylating agents, but not that to MMC (Figure 3E). Comparable analyses were done in the presence or absence of the MGMT inhibitor O^6^-BG. MCF-7 cells pre-incubated with O^6^-BG at a 20 μM concentration also remarkably enhanced the sensitivity to both BO-1055 (Figure 3F) and BCNU (Figure 3G). This suggests that BO-1055 might also introduce lethal O-alkyl DNA adducts in addition to DNA-ICL, and that BO-1055 possesses both types of DNA alkylating activities, which may help to delay chemoresistance in clinical applications.

### Inhibition of MGMT enhances the BO-1055-induced DNA damage response

**Figure 4:**
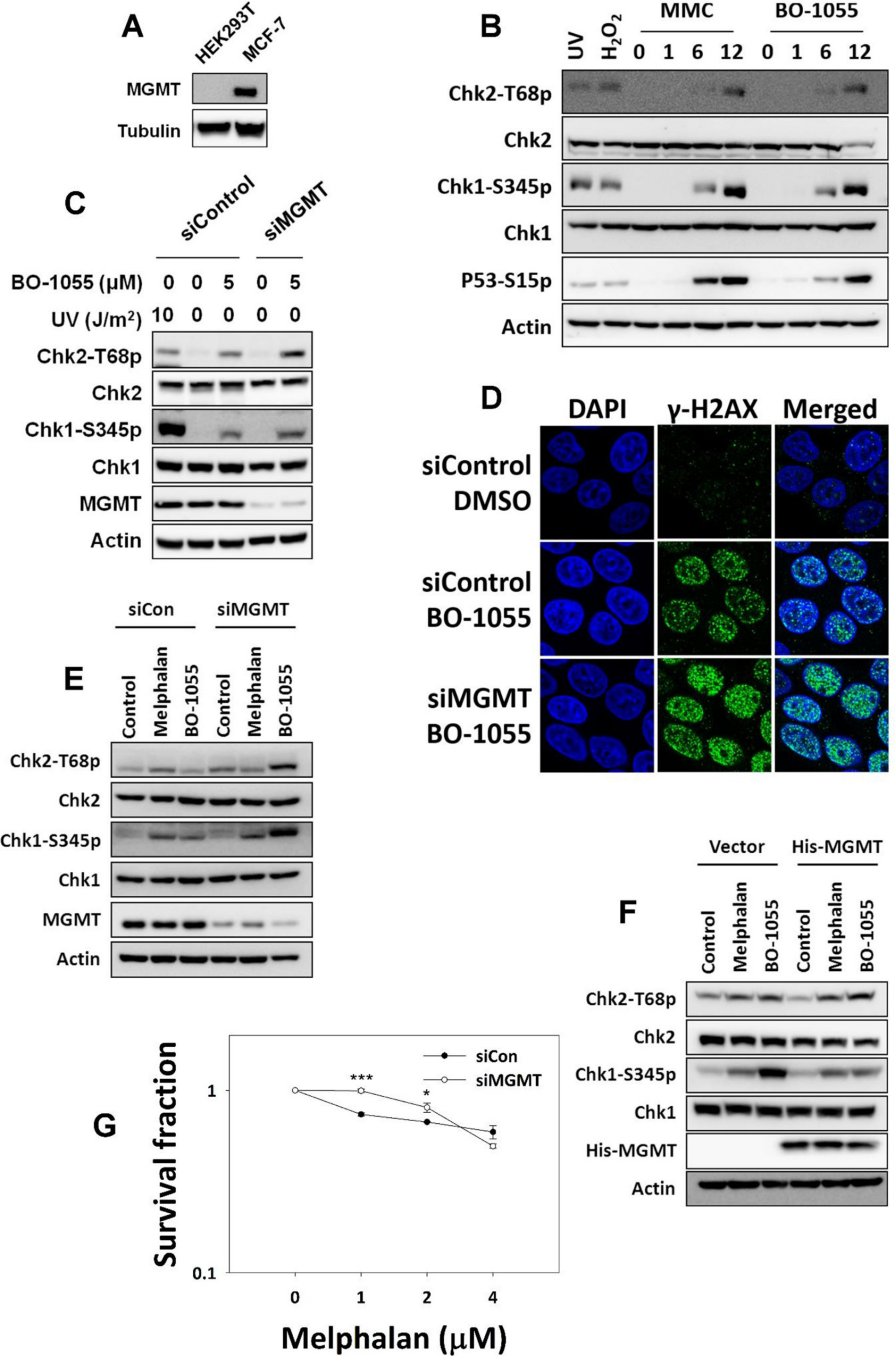
MGMT-mediated repair is required to repair BO-1055-induced, but not melphalan-induced, lesions **A.** Immunoblot analysis showing endogenous MGMT expression in cells. **B.** DDR assessed by detecting the phosphorylation of Chk1 Ser^345^ (Chk1-S345p), Chk2 Thr^68^ (Chk2-T68p), or P53 Ser^15^ (P53-S15p), following the exposure of HEK293T cells to 5 μM of MMC or of BO-1055 for 0, 1, 6, or 12 hours. **C.** DDR induced by BO-1055 in MGMT knockdown MCF-7 cells. **D.** Immunohistochemical staining of the DNA damage marker γ-H2AX (green) and the nucleus DAPI (blue) in MCF-7 cells cultured with siRNA knockdown of MGMT, followed treatment with or without 5 μM of BO-1055 for 24-h. **E.** Detection of DDR in MCF-7 cells transfected with control siRNA or siRNA knockdown of MGMT, following treatment with or without 5 μM of melphalan or 5 μM of BO-1055 for 6-h. **F.** Detection of DDR in HEK293T cells transfected with a control vector or an MGMT expression vector, following treatment with or without 5 μM of melphalan or 5 μM of BO-1055 for 6-h. **G.** *In vitro* clonogenic survival of MCF-7 cells with knockdown of MGMT by siRNA, in MCF-7 cells exposed to the indicated doses of melphalan for 6-h.

As DNA O-alkyl base lesions are mutagenic and harmful to cells, the inhibition of MGMT should trigger the DDR to retard cell cycle progression. As the DDR induced by BO-1055 was found to be lower than that induced by MMC, as shown in MCF-7 cells in Figure 2B, we expected that different MGMT level in cells would lead to differential BO-1055-induced DDRs. To test the impact of the MGMT repair activity on the DDR, we treated low MGMT-expressing HEK293T cells with BO-1055 (Figure 4A) and found that, unlike MCF-7 cells, the DDR induction levels by BO-1055 and MMC were comparable in HEK293T cells (Figure 4B), suggesting that MGMT downregulation increases the cellular response to BO-1055 damage. In high MGMT-expressing MCF-7 cells, decreasing the MGMT expression significantly modified the ATM/ATR-mediated DDR, in which the Chk1 and Chk2 phosphorylation levels (Figure 4C) and the γ-H2AX nuclear foci formation (Figure 4D) induced by BO-1055 were increased. These findings support that BO-1055 might introduce lethal O-alkyl adducts on DNA (Figure 3C and 3F), which can be repaired by MGMT. By contrast, when treating cells with melphalan, which is one of the derivatives of N-mustard for clinical use in treating cancers, the drug-induced DDR was not enhanced in MCF7 cells that had been transfected with MGMT siRNA (Figure 4E). Overexpression of MGMT in HEK293T cells suppressed the BO-1055-induced, but not the melphalan-induced, DDR (Figure 4F). The survival effect of MGMT knockdown in MCF-7 cells to different doses of melphalan treatment was uncertain (Figure 4G). These data suggests that MGMT participates in mediating the BO-1055-induced DDR in our system. This in turn indicates that BO-1055 can produce O-alkyl base lesions and can possibly be repaired by MGMT. However, melphalan like BO-1055 belongs to N-mustard compounds, but seems unlikely to produce O-alkyl adducts on DNA.

### Checkpoint inhibitors enhance BO-1055 sensitivity

**Figure 5:**
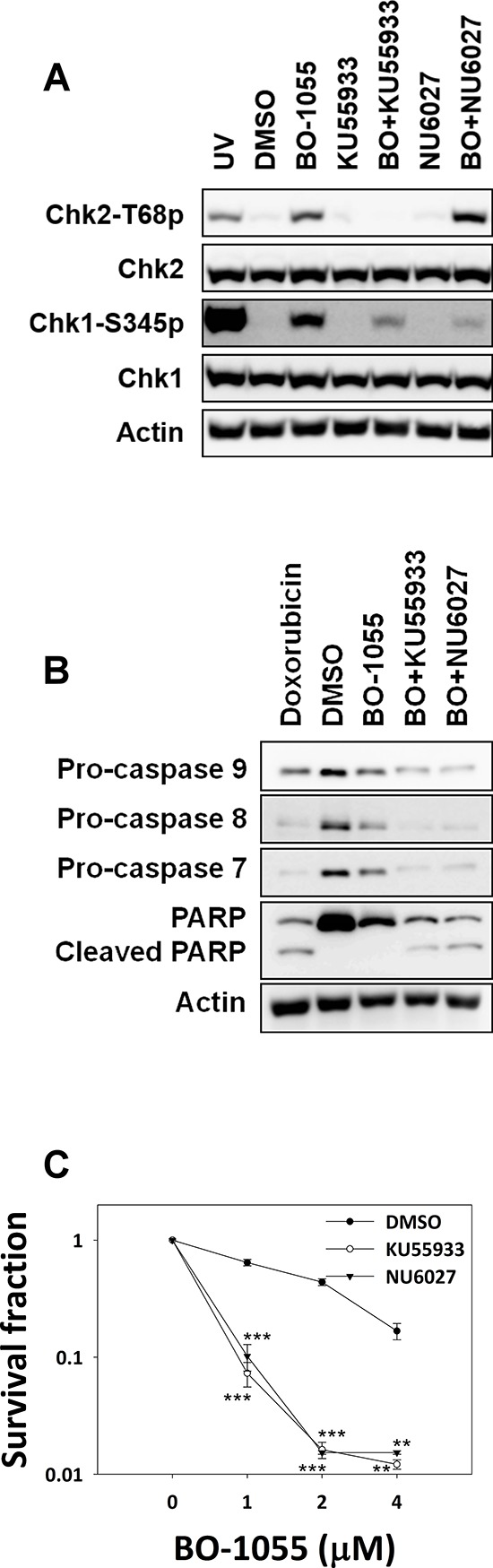
Inhibitors of ATM or ATR enhance the sensitivity of MCF-7 cells to BO-1055 **A.** Immunoblot analysis showing DDR in MCF-7 cells with or without exposure to 5 μM of BO-1055 alone, or co-treatment with 10 μM of NU6027 (BO+NU6027) or 10 μM of KU55933 (BO+KU55933) for 6-h. **B.** Immunoblot analysis showing cell death, assessed by detecting the expression of pro-caspase-7, pro-caspase-8, pro-caspase-9, or PARP following the exposure of MCF-7 cells to 5 μM of BO-1055 alone, or with co-treatment with 10 μM of NU6027 or 10 μM of KU55933 for 72-h. **C.** *In vitro* clonogenic survival of ATM or ATR activity inhibition in MCF-7 cells, by pre-treatment with 10 μM of NU6027 or 10 μM of KU55933 for 30 min, followed by exposure to 5 μM of BO-1055 for 6-h.

Tumors have the ability to modify their repair capacities through a variety of mechanisms, in order to survive chemotherapy [28]. Inhibition of DNA-damage checkpoints is a promising strategy in the sensitization of cancers to chemotherapy; thus, we next investigated the effects of checkpoint kinase inhibition on BO-1055 sensitivity. Pharmacologically, the pretreatment with 10 μM of the ATM inhibitor KU55933 [29] or the ATR inhibitor NU6027 [30] clearly inhibited BO-1055-induced Chk2 and Chk1 phosphorylation, respectively (Figure 5A). The checkpoint suppression led to the cleavage of procaspases and PARP1, as well as to a significant increase in MCF-7 cell sensitivity, when treated with BO-1055 combined with KU55933 or NU6027 (Figure 5B and 5C). Furthermore, BO-1055 sensitivity was also increased in cells by applying a very low concentration of WYC0209 (Supplementary Figure S4), which is an ATR-specific inhibitor that downregulates Chk1 phosphorylation and FANCD2 mono-ubiquitination, in response to DNA damage [31]. Therefore, BO-1055 was confirmed to induce the ATM/ATR-mediated DDR, and simultaneously inhibits either of checkpoints to further increase cell sensitivity to BO-1055 treatment. While the *in vitro* data is convincing, an *in vivo* xenograph model would be more compelling evidence to suggest that combining BO-1055 and ATM/ATR inhibitors effectively decreases the survival of cancer cells.

## DISCUSSION

### BO-1055 is a DNA-ICL agent targeted to proliferating cells

To overcome the insufficiency of clinically used DNA alkylating agents, we previously designed and synthesized various types of DNA-directed alkylating agents, which displayed good pharmacokinetic profiles. However, these conjugates are lipophilic and have poor water solubility. Therefore, we recently prepared a series of novel water-soluble N-mustard-benzene conjugates bearing a urea linker. The benzene ring contains a variety of hydrophilic side-chains (tertiary amino functions), which enable the formation of water-soluble acid salts [19]. Of these agents, the BO-1055 compound was found to have a broad spectrum of antitumor activity and potent therapeutic efficacy against human MX-1 (breast cancer), PC3 (prostate cancer), HCT-116 (colon cancer), and U87 (glioma) cell lines in tumor xenograft models. In this study, we investigated the effects of BO-1055 on DNA lesions and the DNA repair system at the molecular and cellular levels. DNA repair genes are the caretakers of the genome. They have been recognized as tumor suppressors and associated with the therapeutic outcome of anticancer agents [32]. As a consequence of lack in timely completion of DNA repair, severe DNA lesions would lead to cell death. Therefore, the lesion spectrum and repair mechanisms of BO-1055 could be examined by comparing the drug sensitivity among cells with different levels of expression of DNA repair genes. On the other hand, BO-1055 and MMC treatment can cause both apoptotic-like and necrotic-like death, depending on the drug concentration, assessed by annexin V/PI living staining, such that the time required to increase the polyploidy nuclei cells is parallel to that required to increase the PI permeable cells. This implies that MMC and BO-1055 induce fatal polyploidy leading to necrotic-like death. The necrotic-like death of cells might reflect that mitotic catastrophe was significantly elevated following treatment with high doses of MMC or BO-1055. As with MMC, our results suggest that BO-1055 has a selective sensitivity toward highly proliferative cancer cells.

### BO-1055 produces O-alkyl adducts in addition to N-alkyl adducts

In this study, we found that BO-1055 induces FANCD2 mono-ubiquitination reflecting the induction of DNA-ICL lesions. Like MMC damage, when the expression of the HR proteins such as ATM, Chk2, or Rad51, or the NER protein XPG were respectively decreased, it led to the sensitization of MCF-7 cells to BO-1055 treatment. We observed that MMC treatment increased the S-phase population and led to a following increase in highly aberrant DNA content in MCF-7cells, suggesting that MMC produces ICL leading to replication stress and improper chromosome segregation. BO-1055 also caused replication stress but did not appear in high DNA content in cell populations at same concentration. This reflects that only a portion of BO-1055 forms ICL damage at low concentrations, relative to MMC, and that it was trapped during replication, together with the other forms of damage. Of these types of modifications, O-alkylated DNA bases will be recognized due to mispairs, and ATR/Chk1 checkpoints will be activated during DNA replication [33]. Our results suggests that the intensity of DDR induced by BO-1055 correlates to its MGMT expression status; BO-1055 induced DDR at a lower intensity than MMC in high MGMT-expressing MCF-7 cells, but induced the DDR at the same intensity in low MGMT-expressing HEK293T cells. This implies that the BO-1055 induction of DDR at a lower intensity occurs because a proportion of BO-1055 lesions can be repaired rapidly and efficiently in MGMT-expressing MCF-7 cells. In other words, BO-1055 might produce O-alkyl adducts which can be recovered by MGMT, but not N-alkyl adducts that are recovered by the ABH2- and MPG-dependent pathways.

### Comparison with other nitrogen mustards

Biochemical studies have shown that melphalan predominantly causes N-alkylpurine mono-adducts, result in DNA-ICL [34, 35]. Evidence from cell based assays has validated that the NER genes are involved in the removal of melphalan-induced N-alkyl DNA adducts [12–14]. In addition, melphalan resistance was positively correlated with an increase in HR and FA protein expression levels [15–17], suggesting that melphalan produces toxic ICL damage and that cells might become resistant to melphalan when they have acquired an excessive repair capacity. Our results are consistent with previous reports that MGMT protein expression levels do not alter melphalan sensitivity [36, 37]. This confirms that the O-alkyl DNA adducts might rarely be produced by melphalan. Overexpressing MGMT in low MGMT-expressing HEK293T cells predominantly decreases BO-1055-induced, but not melphalan-induced, Chk1 phosphorylation, showing the difference in the mechanism of action between BO-1055 and melphalan, and suggesting that BO-1055-insulted cells might carry O-alkyl adducts into the DNA replication phase, which is sensed by the ATR/Chk1 checkpoint [10, 33]. From a repair system point of view, the types of melphalan-induced DNA damage are similar to MMC, but not to BO-1055. Our results demonstrate that BO-1055, like melphalan, produces lethal N-alkyl adducts and cross-linking damage to DNA, which are repairable through the NER and HR pathways. Besides, BO-1055 might additionally produce lethal O-alkyl adducts on DNA, which is repairable by MGMT. Our result suggest that the action of BO-1055 is similar to that of BCNU, but not to that of melphalan, showing that MGMT involves in the repair of lesions. Although there is no evidence to support the removal of a bulky adduct on O^6^-guanine by MGMT, MGMT can recognize differential alkylation on the O^6^ position of guanine [38–40]. As the multiplicity of genotoxic adducts might be produced by N-mustards, continuous biochemical study of the precise interaction between BO-1055 and DNA is particularly important to understand its mechanism of action.

### ATM and ATR inhibitors are backup stratagems to improve BO-1055 sensitivity

DNA repair genes are frequently affected in tumors, and become diagnostic markers to predict the tumor response to chemotherapy [41–45]. Our study clearly suggests that BO-1055 may be effective in the treatment tumors with dysfunctional FA, NER, HR, or MGMT proteins. Nevertheless, we assume that, as with most chemotherapeutic agents, BO-1055 might have an effective initial response but eventually be met with an acquired resistance in complex tumors. Fortunately, when a DNA-damaging agent requires multiple repair routes to fix the damaged DNA, the time to develop resistance to chemotherapy will be delayed. The requirement of multiple repair systems is precisely a distinguishing feature that renders BO-1055 beneficial for clinical use. As previous reports have revealed that checkpoint inhibitors can improve sensitivities toward DNA-damaging agents [46, 47], targeting the drug responsive checkpoint kinases is an effective strategy to overcome BO-1055 resistance. We found that the sensitivity of cancer cells to BO-1055 was increased following a combined treatment with the inhibitors of the DNA damage sensors ATM and ATR kinases, suggesting that both ATR and ATM are important in the repair of BO-1055-induced lesions in different fashions. According to previous reports, ATM can be activated during the DSBR [22], NER [48] and MGMT repair [49] processes against lethal alkylating damage, but the ATM inhibitor does not improve temozolomide sensitivity when the tumor highly expresses MGMT [50], this implies that temozolomide produces relatively low amount of ICL than MGMT repairable O-alkyl adducts on DNA. The ATR-dependent FA repair pathway is required to repair DNA-ICL damage [51, 52], and ATR and FA protein overexpression were found in melphalan resistance, which can be overcome with an ATR inhibitor, but not an ATM inhibitor [16, 53]. Here, ATR and ATM indeed cooperated in their response to chemotherapeutics in different tumor contexts. Further studies, including xenograft animal test, will be helpful to unravel mechanism underlying BO-1055 resistant and to make decisions in the selection of checkpoint inhibitors, to improve BO-1055 sensitivity in secondary tumors.

In conclusion, chemotherapy is recommended as first-line treatment in many tumors. The responsiveness to chemotherapeutics in the clinic will not last because of tumor heterogeneity driven by intrinsic and extrinsic factors. Therefore, the continuous development of chemotherapeutic agents is critical due to the diversity of tumors. DNA damage-based checkpoints and repair activity determines the fate of cells to chemotherapy. Our informative data on BO-1055 in this system offers insights into the clinical implications of this compound in personalized tumor therapy.

## MATERIALS AND METHODS

### Cell culture and chemicals

Cell lines were purchased from the Bioresource Collection and Research Center (BCRC), Hsinchu, Taiwan) and maintained in cell culture media (Sigma-Aldrich) supplemented with 10% fetal bovine serum (Gibco). HEK293T human embryonic kidney cells (BCRC 60019) and MCF-7 human breast cancer cells (BCRC 60436) were maintained in DMEM. Chinese hamster ovary (CHO) cells AA8 (BCRC 60126), EM9 (BCRC 60500), and UV24 (BCRC 60175) were maintained in MEM. BO-1055 was synthesized as previously described [19]. Alkylating agents, including methyl methanesulfonate (MMS), MMC, BCNU and melphalan, inhibitors O^6^-benzylguanine (O^6^-BG), NU6027 and NU7441, as well as DNA strand breaks agent doxorubicin, were purchased from Sigma-Aldrich. KU55933 was purchased from Tocris Bioscience. For DDR induction, BO-1055 or MMC was added to the culture medium for the indicated time period before cells were harvested. Cells irradiated with UV damage (CL-1000; UVP) at 10 J/m^2^ were served as DDR positive controls.

### RNA interference

The information on the siRNAs used in the study is listed in Supplementary Table S1. A final concentration of 20 nM was achieved for each gene specific siRNA and scrambled RNA (scRNA) used in cell transfection, which was performed with Lipofectamine2000 (Invitrogen), according to the manufacturer's instructions.

### Antibodies and western blotting

Protein extraction and western blot assays were performed as previously described [31]. The information on the primary and secondary antibodies used in the study is listed in Supplementary Table S2. Primary antibodies were recognized by HRP-coupled secondary antibodies, and developed by Immobilon™ Western (Millipore). The images of non-saturated bands were captured using a luminescent image analyzer (LAS-4000 mini; Fujifilm).

### Immunofluorescence

MCF-7 cells were seeded on coverslips 1 day prior to drug treatment, and incubated in a culture medium containing 0.1% DMSO or with drugs at the indicated concentration for 24-h, followed by fixation in 2% paraformaldehyde in DMEM at 4°C overnight. Cells on coverslips were then briefly rinsed with PBS and permeabilized with 0.5% Triton X-100 for 10 min, before being stained with a primary antibody against γ-H2AX (clone JBW301; Merck-Millipore). Cells on coverslips were then incubated with an Alexa Fluor 488-conjugated secondary antibody, followed by the application of 1 μg/mL of DAPI for nuclear counterstaining. Nuclear fluorescence images were captured by confocal laser scanning microscopy (FluoView FV1000; Olympus) using the same parameter settings.

### Flow cytometry

Briefly, MCF-7 cells that had been cultured overnight in a 60-mm dish were treated with the indicated concentration of BO-1055 for different time periods. To evaluate the cell-cycle distribution, the cells were harvested and fixed with 95% methanol for 2 h. They were then rehydrated in 1 × PBS buffer before the DNA was stained with 10 μg/mL of propidium iodide (PI; Invitrogen), with 100 μg/mL of RNase A (Sigma-Aldrich) at room temperature for 30 min; the cells were protected from light exposure throughout this procedure. The PI intensity, reflecting the DNA content, was analyzed using a LSR II Flow Cytometer (BD Biosciences) and FlowJo software (FlowJo LLC). For the measurement of apoptosis, the harvested cells were immediately stained with annexin V-FITC and PI, and subsequently analyzed by an Accuri C6 flow cytometer (BD Biosciences).

### Clonogenic survival assay

48-h after siRNA transfection, a portion of the cells was harvested and reseeded to perform the clonogenic survival assay, and the remainder was collected for western blot, to confirm the gene-silencing efficiency. To evaluate the ability of a single cell to survive DNA-damaging treatment, 200 cells were seeded in 6-well plates one day prior to drug treatment, and then replaced in the culture medium containing the 0.1% DMSO vehicle control or the drugs at the indicated concentration for 6-h, followed by washing with 1 × PBS buffer. The treated cells were then cultured in fresh medium for an additional 10 days. The surviving cells formed colonies and were visualized by 0.1% crystal violet staining. Images were captured using a CCD camera (LAS-4000 mini; Fujifilm) and analyzed with the Colony V1.1 software (Fujifilm).

### Data statistics

Data statistics were calculated from three independent experiments. The significant difference for surviving fractions between siRNA/inhibitor treated and control groups in the same dosages of the DNA damaging agent were compared using Student's *t* test. The significant differences of cell cycle distribution and cell death from FACS results were compared using one-way ANOVA with Dunnett's post hoc test. All data were expressed as the mean ± standard error of the mean (SEM). The statistical significance was represented with an asterisk for *p* values < 0.05 and two asterisks for *p* values < 0.01, and three asterisks for *p* values < 0.001.

## SUPPLEMENTARY FIGURES AND TABLES


