# Supplementary material for: Repairing of N-mustard derivative BO-1055 induced DNA damage requires NER, HR, and MGMT-dependent DNA repair mechanisms
Source: Oncotarget. 2015 Jul 17;6(28):25770–83. doi: 10.18632/oncotarget.4514 (PMC4694865; doi:10.18632/oncotarget.4514)
Supplement: Supplementary file 1 [file oncotarget-06-25770-s001.pdf]

SUPPLEMENTARY FIGURES AND TABLES

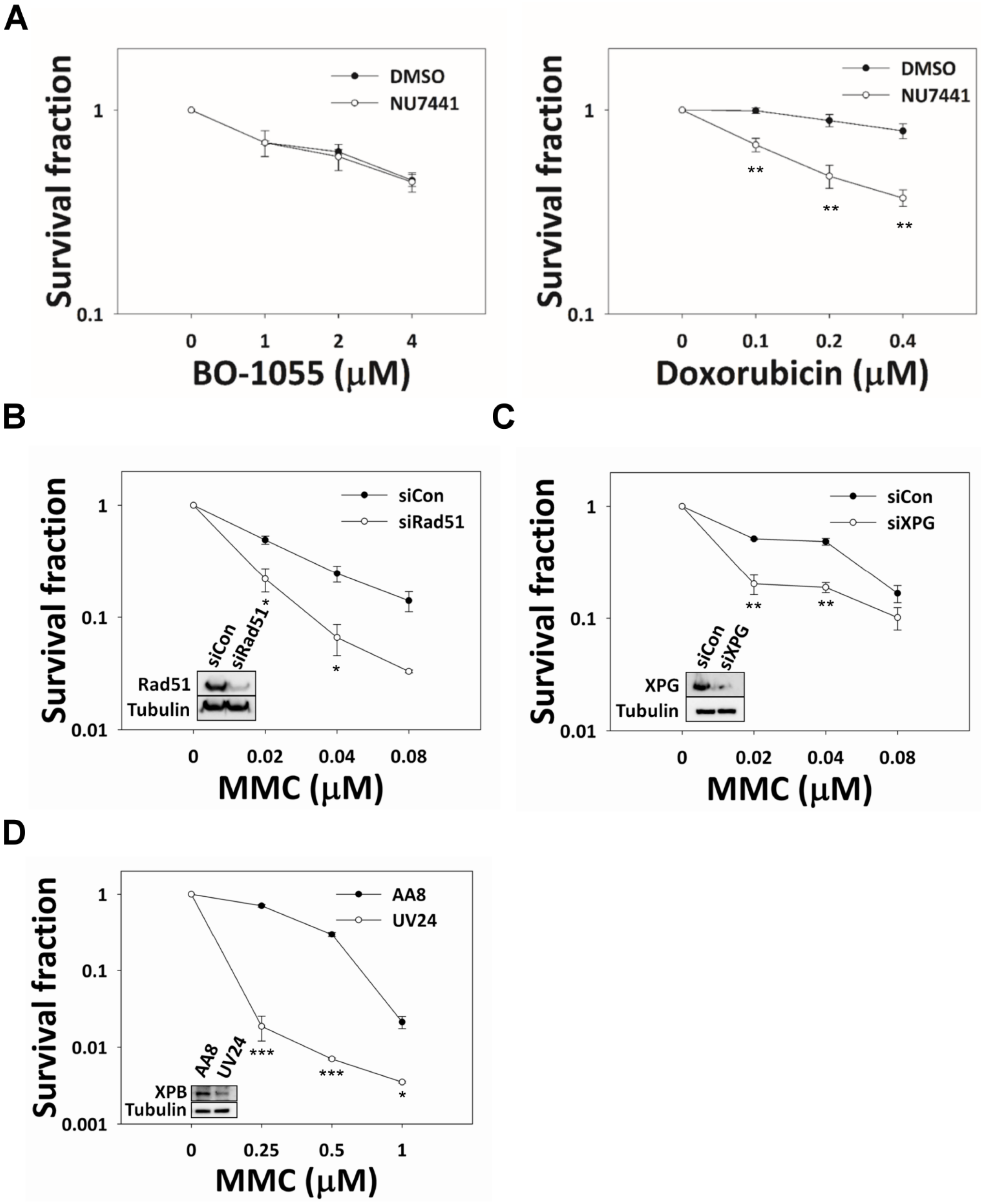

Supplementary Figure S1: Involvement of HR and NER genes in drug sensitivity. *In vitro* clonogenic survival of MCF-7 cells with DNA-PKcs inhibitor NU7441 **A.** or knockdown of Rad51 **B.** or XPG **C.** by siRNAs, or of *XPB*-defective UV24 CHO cells **D.** exposed to the indicated doses of BO-1055, doxorubicin, or MMC for 6-h. The immunoblots embedded in the clonogenic survival plots show the efficiency of gene knockdown for each individual experiment.

A

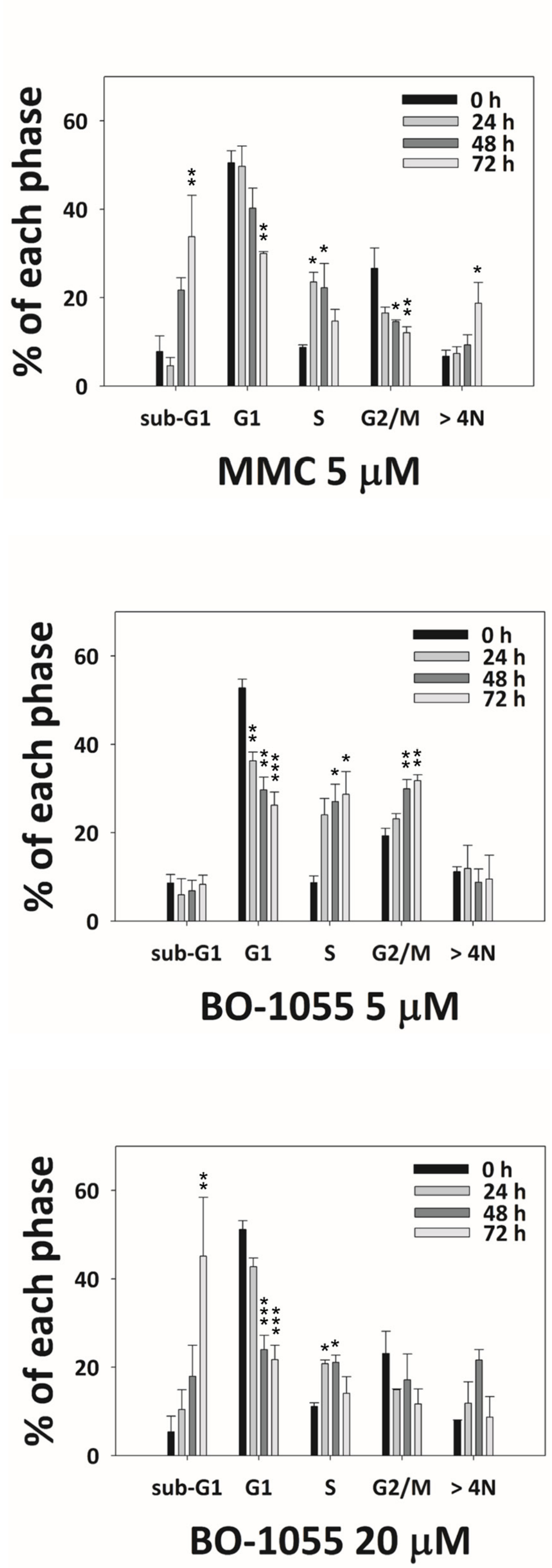

Supplementary Figure S2: FACS analysis for cell-cycle distribution and cell death. A. Quantitative results of FACS histogram analysis of DNA content by Figure 2D. (*Continued*)

B

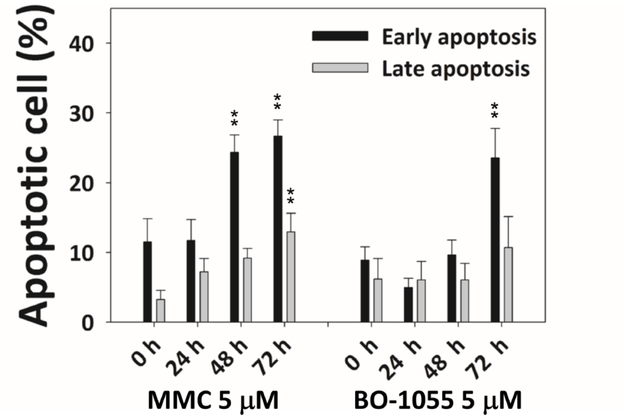

C

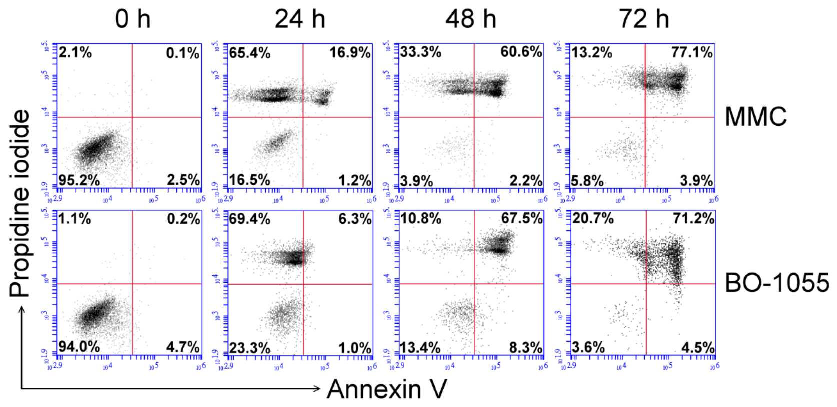

Supplementary Figure S2: (*Continued*) FACS analysis for cell-cycle distribution and cell death. B. Quantitative results of FACS dot-blot analysis for cell death by Figure 2E. The experiment of (A) and (B) were performed three times, and the quantitative results expressed as the mean ± SEM. **C.** AnnexinV/PI double staining in living cells was conducted following the exposure of cultured MCF-7 cells to 20 μM of MMC or of BO-1055 for the indicated times.

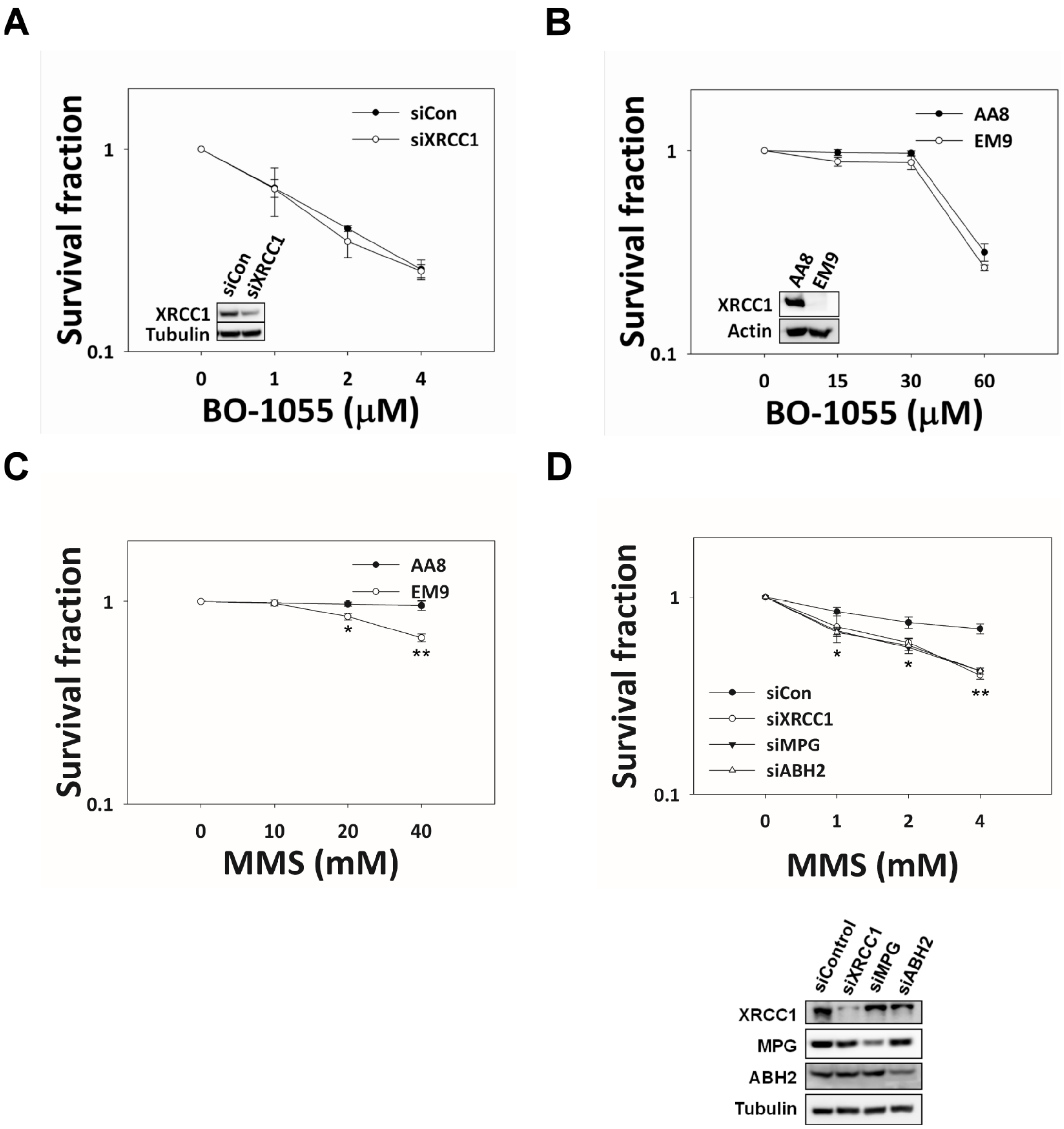

Supplementary Figure S3: *In vitro* clonogenic survival assay. MCF-7 cells with knockdown of XRCC1 **A.** or *XRCC1*-defective EM9 CHO cells **B.** exposed to the indicated doses of BO-1055 for 6-h. MMS sensitivity assayed in *XRCC1*-proficient AA8 or *XRCC1*-defective EM9 CHO cells **C.** or in MCF-7 cells with knockdown of XRCC1, MPG, or ABH2 by siRNAs **D.** The immunoblots embedded in the clonogenic survival plots show the efficiency of gene knockdown for each individual experiment.

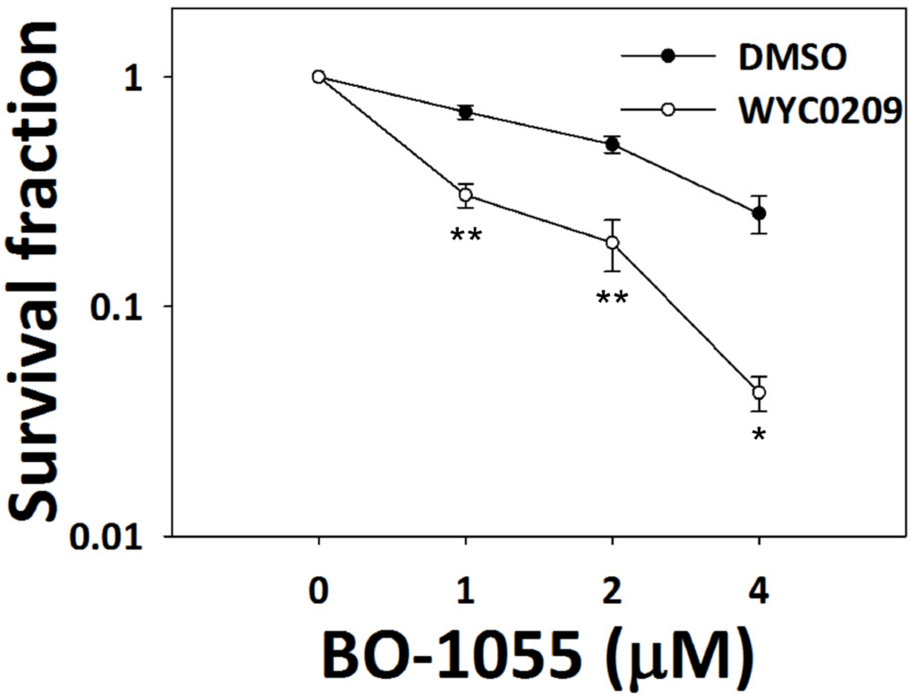

Supplementary Figure S4: ATR inhibition by WYC0209 enhances BO-1055 sensitivity. *In vitro* clonogenic survival assay of MCF-7 cells, following inhibition of ATR activity by 20 nM of WYC0209, with exposure of the MCF-7 cells to the indicated doses of BO-1055 for 6-h.

Supplementary Table S1: Target sequences of siRNA oligos

Gene Name	Accession	Target sequence
XPG	NM_000123	ACCAAGCACTTAAAGGAGTCC
ATM	NM_000051	AAGCGCCTGATTCGAGATCCT
Chk2	NM_007194	AATGTGTGAATGACAACTACT
Rad51	NM_133487	GAAGCTGGATTCCATACTGTG
DNA-PKcs	NM_006904	GATCGCACCTTACTCTGTTGA
XPB	NM_000122	GACTTCTTGGTGGCTATTGCA
XRCC1	NM_006297	AACTCGACTCACTGTGCAGAA
ABH2	NM_001145374	GACAGACCTTCAACTTTGTGCTCAT
MPG	NM_002434	AAGAAGCAGCGACCAGCTAGA
MGMT	NM_002412	CTGCACGAAATAAAGCTCCTG
Control	None	AAGTCAATATGCGACTGATGG

Supplementary Table S2: Primary antibodies used in immunoassays

Targets	Species	Catalog	Distributors
MPG	Mouse mAb (1E10)	H00004350-M04	Abnova
Caspase-7	Mouse mAb (B94-1)	554002	BD pharmingen
XPG	Rabbit pAb	A301–484A	Bethyl
Phospho-p53 (S15)	Mouse mAb (16G8)	9286	Cell signaling
Phospho-Chk2 (T68)	Rabbit mAb (C13C1)	2197	Cell signaling
Phospho-Chk1 (S345)	Rabbit mAb (133D3)	2348	Cell signaling
Chk2	Mouse mAb (1C12)	3440	Cell signaling
Caspase-8	Mouse mAb (1C12)	9746	Cell signaling
XPB	Rabbit pAb (C1C3)	GTX112923	GeneTex
ATM	Mouse mAb (2C1)	GTX70103	GeneTex
Mouse IgG	Alexa Fluor® 488 goat anti-mouse IgG	A-11001	Invitrogn
Mouse IgG	HRP-conjugated goat anti-mouse IgG	115-035-146	Jackson Immunoresearch
Rabbit IgG	HRP-conjugated goat anti-rabbit IgG	111-035-144	Jackson Immunoresearch
MGMT	Mouse mAb (MT3.1)	MAB16200	Millipore
Phospho-histone H2A.X (S139)	Mouse mAb (JBW301)	05-636	Millipore
Chk1	Mouse mAb (G4)	sc-8408	Santa Cruz
Rad51	Rabbit pAb (H92)	sc-8349	Santa Cruz
FANCD2	Mouse mAb (FI17)	sc-20022	Santa Cruz
Caspase-9	Rabbit pAb (F7)	sc-17784	Santa Cruz
PARP1	Mouse mAb (F2)	sc-8007	Santa Cruz
Phospho-ATM (S1981)	Mouse mAb (10H11.E12)	sc-47739	Santa Cruz

(*Continued*)

Targets	Species	Catalog	Distributors
ABH2	Mouse mAb (hABH2–7)	A8228	Sigma-Aldrich
Actin	Rabbit pAb	A2066	Sigma-Aldrich
α-Tubulin	Mouse mAb (DM1A)	T6199	Sigma-Aldrich
XRCC1	Mouse mAb (33-2-5)	MS-434	Thermo Scientific
DNA-PKcs	Mouse mAb Cocktail (Ab-4)	MS-423	Thermo Scientific

Supplementary Table S3: Cytotoxicity of compounds in MCF-7 cells

Compounds	IC_{50} values (µM)
BO-1055	13.92 ± 0.84
MMC	5.74 ± 0.08
BCNU	161.05 ± 1.03
O^6-BG	143.75 ± 7.11

IC_{50} values refer to the concentration needed to induce 50% of cell death following treatment with the indicated compounds. Cells were treated with the compounds at various concentrations ranging from 1–20 µM, or from 1–200 µM, for 48 hours. Cell survival was determined using the MTT assay, and the IC50 values are represented as the means ± SE of three independent experiments carried out in triplicates.
